# Spatial Disparities in Access to Healthcare Professionals in Sichuan: Evidence from County-Level Data

**DOI:** 10.3390/healthcare9081053

**Published:** 2021-08-16

**Authors:** Ning Zhang, Wei Ning, Tao Xie, Jinlin Liu, Rongxin He, Bin Zhu, Ying Mao

**Affiliations:** 1 School of Public Policy and Administration, Xi’an Jiaotong University, Xi’an 710049, China; zhangningati@stu.xjtu.edu.cn (N.Z.); ningwei@stu.xjtu.edu.cn (W.N.); xietao2014077049@stu.xjtu.edu.cn (T.X.); herongxin@stu.xjtu.edu.cn (R.H.); 2 Research Center for the Belt and Road Health Policy and Health Technology Assessment, Xi’an Jiaotong University, Xi’an 710049, China; 3 Centre for Global Infectious Disease Analysis, Department of Infectious Disease Epidemiology, Imperial College London, London SW7 2AZ, UK; 4 School of Public Policy and Administration, Northwestern Polytechnic University, Xi’an 710049, China; jinlinliu89@gmail.com; 5 School of Public Health and Emergency Management, Southern University of Science and Technology, Shenzhen 518055, China

**Keywords:** healthcare professional, health equity, licensed doctors, registered nurses, China

## Abstract

As a southwestern province of China, Sichuan is confronted with geographical disparities in access to healthcare professionals because of its complex terrain, uneven population distribution and huge economic gaps between regions. With 10-year data, this study aims to explore the county-level spatial disparities in access to different types of healthcare professionals (licensed doctors, registered nurses, pharmacists, technologists and interns) in Sichuan using temporal and spatial analysis methods. The time-series results showed that the quantity of all types of healthcare professionals increased, especially the registered nurses, while huge spatial disparities exist in the distribution of healthcare professionals in Sichuan. The local Moran’s I calculations showed that high–high clusters (significantly high healthcare professional quantity in a group of counties) were detected in Chengdu (capital of Sichuan) and relatively rich areas, while low–low clusters (significantly low healthcare professional quantity in a group of counties) were usually found near the mountain areas, namely, Tsinling Mountains and Hengduan Mountains. The findings may deserve considerations in making region-oriented policies in educating and attracting more healthcare professionals to the disadvantaged areas.

## 1. Introduction

Healthcare professionals play an important role in improving population health and promoting universal health coverage [1,2]. Many previous studies have shown the relationship between qualified healthcare professionals and health outcomes [3]. However, huge inequalities exist in the distribution of healthcare professionals in almost all countries, with the disadvantaged regions having limited access to multiple subtypes of healthcare professionals. For example, inequalities in access to healthcare professionals have been reported in many countries, namely the United Kingdom [4], Australia [5], Spain [6], Thailand [7], South Africa [8], Brazil [9], India [10], Indonesia [11], and Cameroon [12].

As the biggest developing country in the world, China is also confronted with geographical disparities in the healthcare professional distribution. A large number of researchers have studied the equity in the distribution of healthcare professionals in China. Anand et al. found huge urban-rural disparities in doctor and nurse distribution among provincial units in China [13]. Liu et al. found that western China is disadvantaged in the allocation of healthcare professionals [14]. Xie et al. found that the healthcare professionals tended to concentrate in richer regions from 2009 to 2015 as assessed by the concentration index [15]. The research of Zhu et al. revealed the equality in the distribution of healthcare professionals among provincial units in China, which was measured with the Theil-L index, gradually improved from 2004 to 2014 [16].

Although a number of previous studies have focused on the spatial disparities in access to healthcare professionals, almost all the studies paid attention to the province-level disparities, little paid attention to the county-level disparities in access to healthcare professionals in China. In any analysis of geography, the research scales matter greatly. A provincial research scale may thus obscure the diversity of its components (cities, counties). There is no doubt that a smaller research scale contributes exponentially to the applicability and significance of this topic. In addition to that, many studies just emphasized the spatial accessibility of healthcare, which mainly was related the quantity of healthcare, population density and the distance between healthcare services and community; few analyzed the spatial disparities form the perspective of cluster characteristics and variation change. The former kind of research could be regarded as the evidence for site selection of healthcare services, while the latter kind of studies could support the policies of spatial disparities. Additionally, few studies compared the spatial distribution patterns of different subtypes of healthcare professionals.

To fill the research void, this study takes Sichuan, a western province with a number of ethnic minorities, as an example to explore the county-level spatial disparities in access to healthcare professionals. Ethnic minorities, due to historical reasons, are more concentrated in remote and under-developed areas, which thus deserves more attention on the access to healthcare professionals [17]. First, a temporal analysis of the number of healthcare professionals per 1000 of the population in 183 counties in Sichuan will be conducted and visualized. Second, the spatial distribution and spatial clusters of healthcare professionals in 183 counties will be explored and mapped. Third, the temporal and spatial distribution characteristics of different subtypes of healthcare professionals (licensed doctors, registered nurses, pharmacists, technologists and interns) will be compared and discussed. We hope that this study could provide evidence for promoting the equitable distribution of healthcare professionals in Sichuan.

## 2. Materials and Methods

### 2.1. Study Area

**Figure 1:**
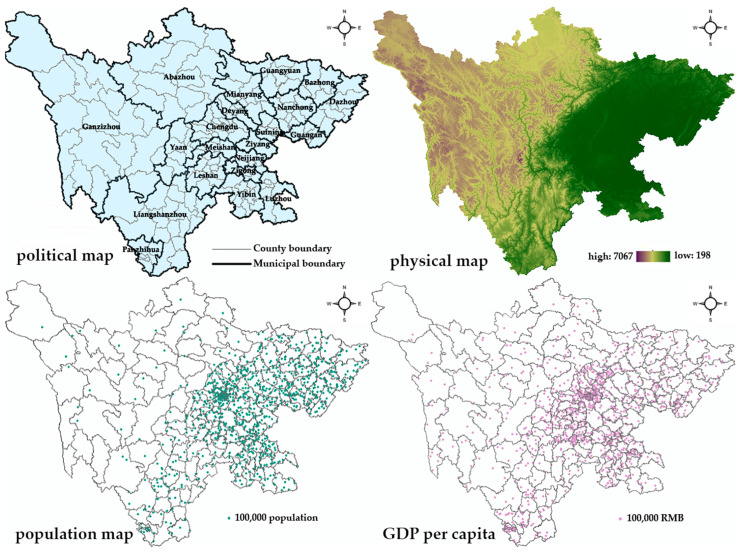
The map of Sichuan. The source of shapefiles was a public database, National Nature Resources and Geospatial basic information database of PRC (http://www.geodata.cn/) (accessed on 15 August 2021). Those shapefiles were under license without need for permission.

Sichuan province has an area of 486,000 km^2^ and a population of 83.41 million in 2018, with Chengdu as its capital. Sichuan province is located in southwestern China; there are 21 municipal-level areas, containing 183 county-level units. Figure 1 shows the administrative, topographic, population and GDP per capita map of Sichuan. Sichuan province has an extremely complex terrain, which spans several geomorphic units, such as the Qinghai-Tibet Plateau, Hengduan Mountains, Yunnan-Guizhou Plateau, Tsinling Mountains, and Sichuan Basin. The basin area is surrounded by various mountains and is at a relatively developed level in the aspects of the economy, education, and medicine. Due to its complex terrain and uneven population distribution, huge economic gaps were reported between regions [18], which help to explain the inequalities in healthcare professional distribution in Sichuan. There are three minority autonomous municipal-level areas, namely Abazhou, Ganzizhou and Liangshanzhou, which includes ethnic minority groups such as the Yi, Tibetan, and Qiang peoples. They are both located in the western part of Sichuan.

### 2.2. Study Setting and Data Resources

The study used the 2009–2019 year-end data from 183 county-level units in Sichuan. The year 2009 is the first year after the new medical reform in China, and various policies were formulated and promulgated after the new medical reform [19]. The data were collected from the Sichuan Health Statistics Yearbook, Sichuan Health and Family Planning Statistical Yearbook, Sichuan Statistical Yearbook published by the Sichuan Provincial Bureau of Statistics; and the China County Statistical Yearbook published by the National Bureau of Statistics of China. Tables S1 and S2 show the number of population and healthcare professionals of county-level units in Sichuan. Table S3 shows the number of healthcare professionals at the municipal level in Sichuan.

We used the number of different subtypes of healthcare professionals per 1000 of the population to measure the accessibility to healthcare professionals. It is an indicator widely recognized by WHO and the Chinese government [20,21]. This study focused on the health technicians (HT), which directly provide healthcare services and thus played an important role. HT can further be divided into five subtypes, i.e., licensed doctors (LD), registered nurses (RN), pharmacists (PH), technologists (TE) and interns (IN) [22].

### 2.3. Time-Series Analysis

The time-series analysis illustrated the temporal trend of the number of healthcare professionals per 1000 people in county-level units. On the one hand, the study time (2009–2019) was divided into two equal periods, namely, 2009–2014 and 2014–2019. The growth rate at the municipal level was calculated and tested by the linear-by-linear association. On the other hand, the box plots were introduced to demonstrate the temporal trends with basic statistics, i.e., the maximum, minimum, median, upper quartile, lower quartile [23,24].

### 2.4. Spatial Clustering Analysis

#### 2.4.1. Spatial Autocorrelation Analysis

Spatial autocorrelation is defined as “indicators negatively or positively associated with themselves over space” [25,26], which means whether the existence of variables in one unit has affected the existence of other units, negatively or positively, more or less. Moran’s I is one of the most widely used indicators for spatial autocorrelation analysis, which has the unique advantage of exploring spatial variations and identifying units or subsets of units that are unusual.

**Figure 2:**
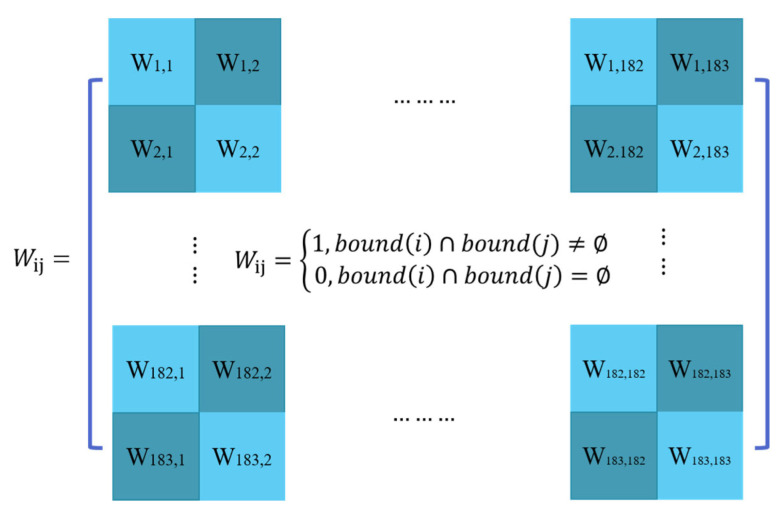
The spatial weight matrix.

To gain insight into the spatial distribution of the healthcare professionals, global Moran’s I, local Moran’s I and relevant graphic tools were introduced to analyze and visualize the spatial autocorrelation results of the number of healthcare professionals per 1000 of the population, which have been applied in many studies. According to Cliff and Ord [27,28], the formula for Moran’s I used in this paper is as follows:

(1) $$\mathrm{Global}\;\mathrm{Moran}’s\;I=\frac{n\sum_{i=1}^{n}\sum_{j=1}^{n}w_{\mathrm{ij}}\left(x_{i}\;-\;\bar{x}\right)\left(x_{j\;}\;-\;\bar{x}\right)}{\sum_{i=1}^{n}\sum_{j=1}^{n}w_{\mathrm{ij}}{\left(x_{i}\;-\;\bar{x}\right)}^{2}}$$

(2) $$\mathrm{Local}\;\mathrm{Moran}’s\;I\;=\frac{\left(x_{i}\;-\;\bar{x}\right)}{m_{0}}\sum_{j}W_{\mathrm{ij}}\left(x_{j}\;-\;\bar{x}\right);\;m_{0}=\sum_{i}{\left(x_{i}\;-\;\bar{x}\right)}^{2}/n$$

where n represents the number of observations; ${\mathrm{xi}}_{}$ denotes the number of healthcare professionals in county i per 1000 people; $x_{j}$ is the number of healthcare professionals in county j per 1000 people; $\bar{x}\;$indicates the mean value of the variable; and $W_{\mathrm{ij}}$ symbolizes a spatial weight matrix of i relative to j. The spatial weight matrix (Figure 2) aggregated all the relations between 183 county-level units, the value of $W_{\mathrm{ij}}$ is determined by boundaries among counties, which is 1 when counties share a boundary and 0 otherwise.

The range of global Moran’s I is from −1 to 1. In this context, a value near −1 represented a spatial aggregation of dissimilarity between values in county-level units, reflecting units with low and high numbers of healthcare professionals per 1000 people gathered together geographically. In contrast, a value near 1 revealed that units with low (or high) numbers of healthcare professionals per 1000 people gathered together geographically. When the value is approximately 0, there is no spatial autocorrelation in the research area, and units with high or low numbers of healthcare professionals per 1000 people in counties are randomly distributed [29,30].

Local Moran’s I was calculated to explore the spatial autocorrelation between each unit and its adjacent units. The range of −1 to 1 is still applicable to local Moran’s I. Based on the results of local Moran’s I, four types of clusters can be detected: namely, high–high (HH), high–low (HL), low–high (LH) and low–low (LL). For example, HL represents a high-value area surrounded by other low-value areas. The other clusters have similar meanings. For both global and local Moran’s I, Monte Carlo randomization (99,999 permutations) was adopted to calculate the significance level.

#### 2.4.2. Space–Time Scan Analysis

The space–time scan statistic was applied to explore the spatial variations in temporal trends. More specifically, we adopt this method is to detect the geographical units that had a significant higher or lower growth of healthcare professionals. The space–time scan was used through a moving circular column [31,32], which scans all the counties throughout the research period. The variations between the inside and the outside of the column were calculated by the log-likelihood ratio (LLR) as follows:

(3) LLR=log{(C/n)^c [((C−c))/((C−n))]^((C−c))}

where *C* represents the total number of healthcare professionals, *c* denotes the observed cases of healthcare professionals inside the window, and n represents the expected number of healthcare professionals workforce inside the window.

The hypothesis would be that there is no difference between the columns by time. According to the LLR, the higher or lower units would be detected. The classification of clusters contained the most likely cluster, secondary cluster, and others. In this study, a Poisson-based analysis was used with Monte Carlo randomization to test the cluster significance [33,34].

**Null** **Hypothesis.** *There is no difference between the columns by time*.

**Alternative** **Hypothesis.** *There is a difference between the columns by time*.

### 2.5. Software Tools

The box plot of time-series analysis of health workforce in Sichuan was visualized by Microsoft Excel (Version office 365, Microsoft Corp., Redmond, WA, USA). SPSS (Version 20, IBM Inc., Armonk, NY, USA) was employed to obtain the Pearson chi-square of linear-by-linear association test. The autocorrelation analysis containing the global Moran’s I and local Moran’s I were calculated through GeoDa (Version 1.8.61, the University of Chicago, Chicago, IL, USA), while the spatial-scan statistics were performed with SatScan (Version 9.5, Kulldorff and Information Management Services, Inc., Boston, MA, USA). Finally, ArcGIS (Version 10.0, ESRI Inc., Redlands, CA, USA) was used to visualize the results.

## 3. Results

### 3.1. Descriptive Analysis

Table S4 presents the number and growth rate of healthcare professionals per 1000 people in each municipal unit, in which the number and growth rate in the two subsequent periods (2009–2014, 2014–2019) can be seen. The bold number represented the growth rate with a significant linear trend, calculated by chi-square, and *p*-value results for the chi-square from the linear-by-linear association test (the results can be seen in Table S5). On the whole, the healthcare professionals displayed a normal and upward trend in most units, while some others revealed unique characteristics. For instance, the number of interns per 1000 people in Nanchong increased by 14.02%, from 0.3113 to 0.5296, in the first period, while a sharp decrease followed from 2014 to 2019, at the rate of −1.12%.

### 3.2. Temporal Trends

**Figure 3:**
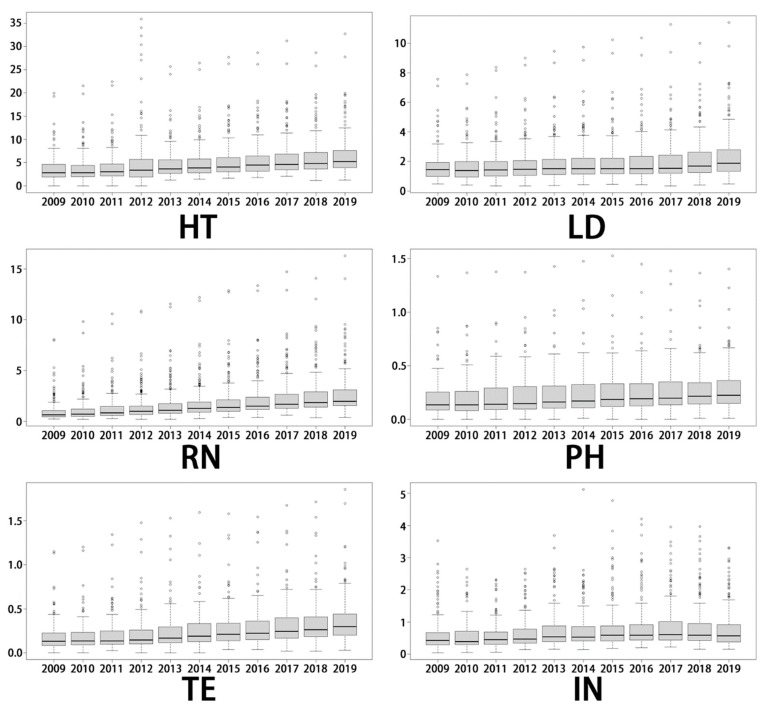
Box plots of the number of different types of healthcare professionals per 1000 people from 2009 to 2019. HT, health technician; LD, licensed doctor; RN, registered nurse; PH: pharmacist, TE: technologist, IN: intern.

Figure 3 uses box plots to display the temporal trend in the number of healthcare professionals per 1000 of the population from 2009 to 2019. The mild and extreme outliers were identified and marked from the examination of the fence points and data. The median and the lower and upper quartiles were marked with a solid line drawn across the box to locate the median. Different healthcare professionals manifested unique characteristics. Regarding the HT, the 25th and 75th percentiles and the median decreased from 2009 to 2010, followed by a slow rise to 2019, while outliers were mainly seen in Chengdu according to calculated data. For LD, the number per 1000 people showed a slow increase in the study period, while the location of the medians gradually went down. The median of RN displayed a gradual upward trend. Similarly, the technologist category experienced an increase as well. Surprisingly, the lowest number of PH per 1000 people remained almost zero in some counties during the whole research period.

### 3.3. Spatial Changing Patterns

#### 3.3.1. Global Spatial Autocorrelation

Table 1 illustrates the results of the global spatial autocorrelation of the healthcare professionals. From the perspective of the *p*-value, all values of global Moran’s I achieved a significance level of 0.001 during the study period. The global Moran’s I of HT experienced a downward trend, followed by a constant decrease from 2016 to 2019, which indicated the weakening agglomeration. For LD, the global Moral’s I remained almost steady from 2009 to 2017, followed by a slight decrease in 2018 and 2019. Similar to HT, the global Moral’s I of RN displayed an overall downward trend. For pharmacists and technologists, global Moran’s I fluctuated during the study period. The global Moran’s I of interns declined dramatically from 0.647758 in 2009 to 0.544234 in 2013; however, this trend reversed, and the global Moran’s I was 0.763703 in 2019.

#### 3.3.2. Local Spatial Autocorrelation

**Figure 4:**
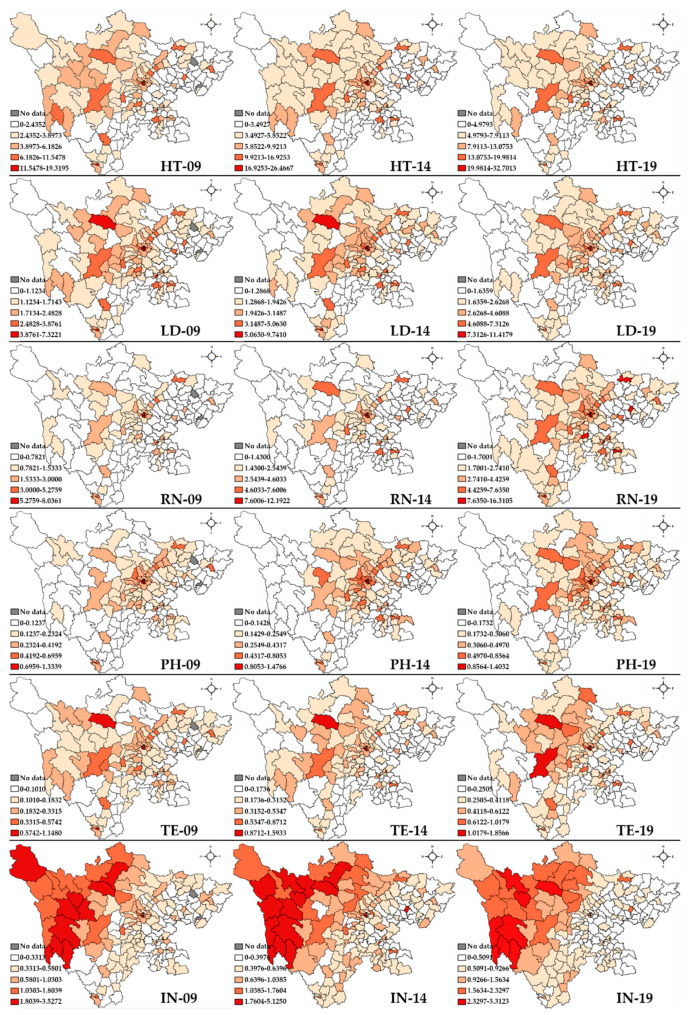
The hierarchy maps of the numbers of all types of healthcare professionals per 1000 people in 2009, 2014, and 2019. HT, health technician; LD, licensed doctor; RN, registered nurse; PH, pharmacist; TE, technologist; IN, intern.

Figure 4 presents the results of local spatial autocorrelation with a hierarchical map of the number of healthcare professionals per 1000 people in 183 counties, which reveals the spatial distribution patterns of healthcare professionals in Sichuan. We classified all the units into five scales by the natural break method, which maximizes the differences between classes. The deeper the red, the higher the number of healthcare professionals per 1000 of the population. In general, fewer healthcare professionals are practicing in the northeastern and southeastern Sichuan, while Chengdu and Panzhihua have relatively rich health human resources.

**Figure 5:**
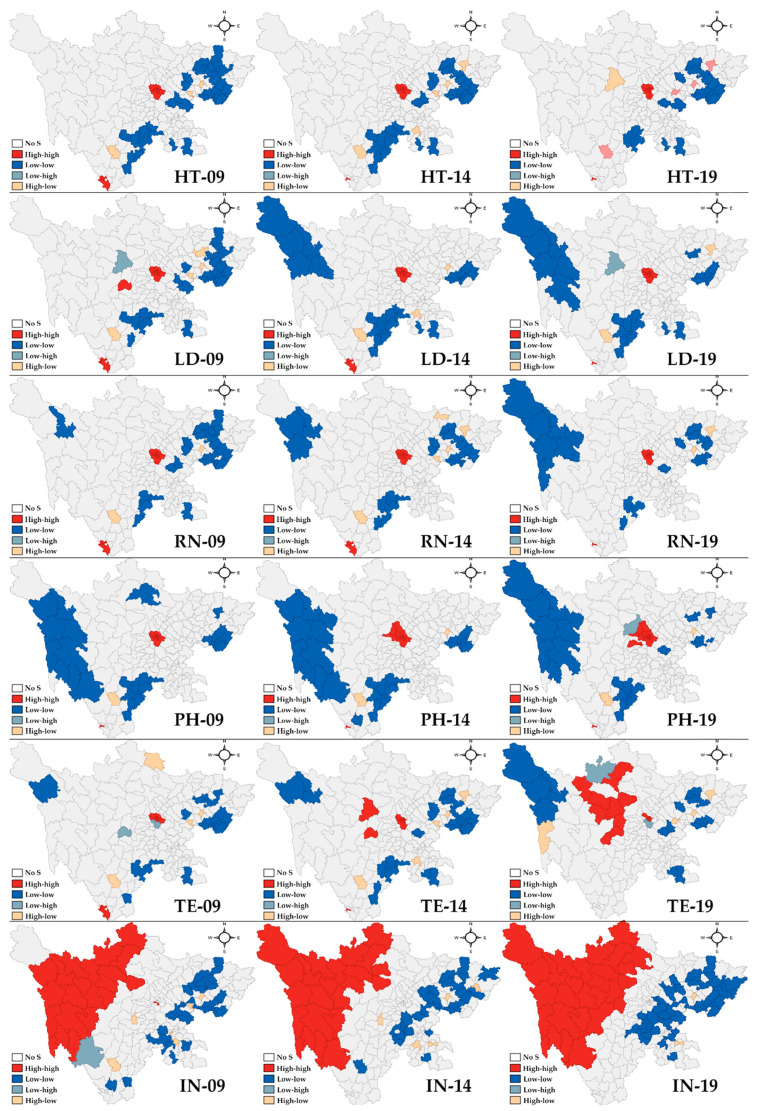
Spatial clusters of all types of healthcare professionals in 2009, 2014, and 2019. HT, health technician; LD, licensed doctor; RN, registered nurse; PH, pharmacist; TE, technologist; IN, intern.

Regarding the spatial changing patterns of health technicians, the spatial clusters of all the healthcare professionals in 2009, 2014 and 2019 are presented in Figure 5. Only local Moran’s I with a significance level below 0.05 was marked with another color on the map. In 2009, most HH clusters were detected in the middle and southern areas (12 counties; municipal level: Chengdu and Panzhihua; topographic feature: Sichuan Basin and valley of Hengduan Mountains). Part of the northeastern and southeastern areas had LL clusters (33 counties; Guangan, Suining, Nanchong, Guangyuan, Ziyang, Leshan, Yaan; Tsinling Mountains and Yunnan-Guizhou Plateau); and HT areas were found in some rich places in Suining, Nanchong and Liangshanzhou (three counties; Tsinling Mountains and Qinghai-Tibet Plateau). The situation in 2014 is similar to that in 2009, except that the HL cluster was also detected in Yibin. In 2019, a county in Abazhou was identified as the LH area.

For LD, 13 counties in the middle area and the southern area, located in Sichuan Basin, the Valley of Hengduan Mountains and Qinghai-Tibet Plateau were identified as HH clusters. The other clusters were similar to those of HT in 2009. In 2014, the LL cluster expanded to the northwestern territories, including Ganzizhou in the Qinghai-Tibet Plateau, while some of the LL clusters in the northeastern area vanished. Furthermore, the northwestern area further expanded. Regarding RN, the whole clustering pattern is similar to that of HT in 2009, except that LL clusters in Ganzizhou were detected early. The number of LL clusters in Ganzizhou gradually increased in 2014 and 2019.

For pharmacists, the HH clusters were found in middle Chengdu and Panzhihua, while LL areas were seen in the Sichuan border area, including 28 counties distributing through Tsinling Mountains, Qinghai-Tibet Plateau, and Yunnan-Guizhou Plateau. Xichang was distributed in the HL cluster. As time goes by, the HH clusters and LL clusters both widened to surrounding places in 2014. In 2019, two specific locations, Abazhou and Ganzizhou, were detected as LH and HL areas, respectively.

Considering TE in 2009, HH clusters were in Chengdu (11 counties, Sichuan Basin), while LL clusters could be detected in northeastern, northwestern and southeastern territories, containing 18 counties in Tsinling Mountains, Qinghai-Tibet Plateau, and Yunnan-Guizhou Plateau. HL clusters were found in Suining, Nanchong, Abazhou, and Liangshanzhou, while LH clusters could be seen in Meishan and Yaan (bordering the rich region). In 2014, a new HH cluster was discovered in one county in Abazhou, which expanded to other counties in 2019. A new LH cluster was observed in Abazhou as well.

In contrast to other healthcare professionals, interns experienced a unique change. In 2009, the western and middle area had HH clusters (23 counties; Ganzizhou, Abazhou, and Chengdu; Sichuan Basin and Qinghai-Tibet Plateau). The LL clusters could be seen in Guangyuan, Bazhong, Nanchong, Suining, Guangan, Liangshanzhou, Leshan, Yibin and Luzhou (25 counties, Tsinling Mountains and Yunnan-Guizhou Plateau). HL clusters were located in some rich places in Nanchong, Ziyang, Yaan, and Liangshanzhou, with LH clusters in Liangshanzhou as well. As time passed, the HH and LL clusters expanded.

### 3.4. Spatial Changing Patterns

**Figure 6:**
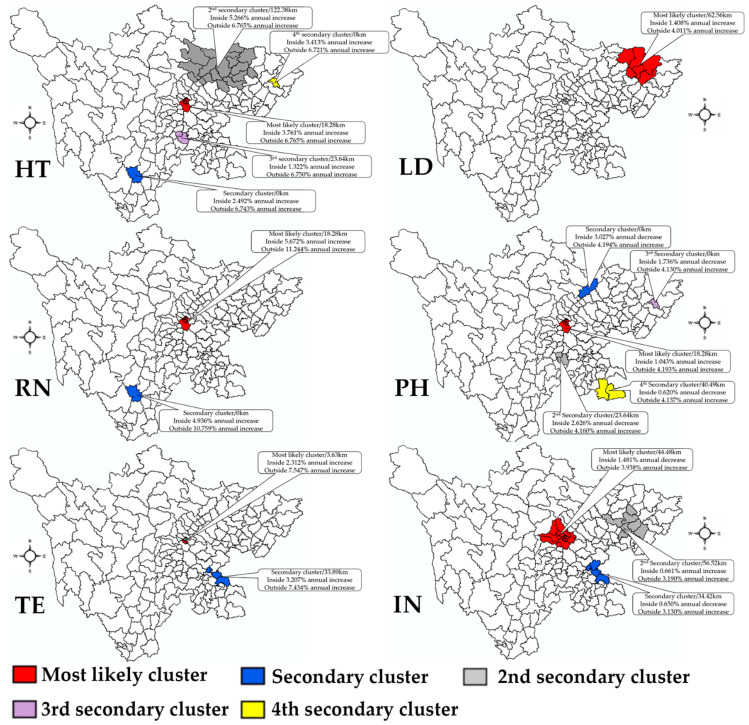
Lower spatial variation in temporal trends. HT, health technician; LD, licensed doctor; RN, registered nurse; PH, pharmacist; TE, technologist; IN, intern.

**Figure 7:**
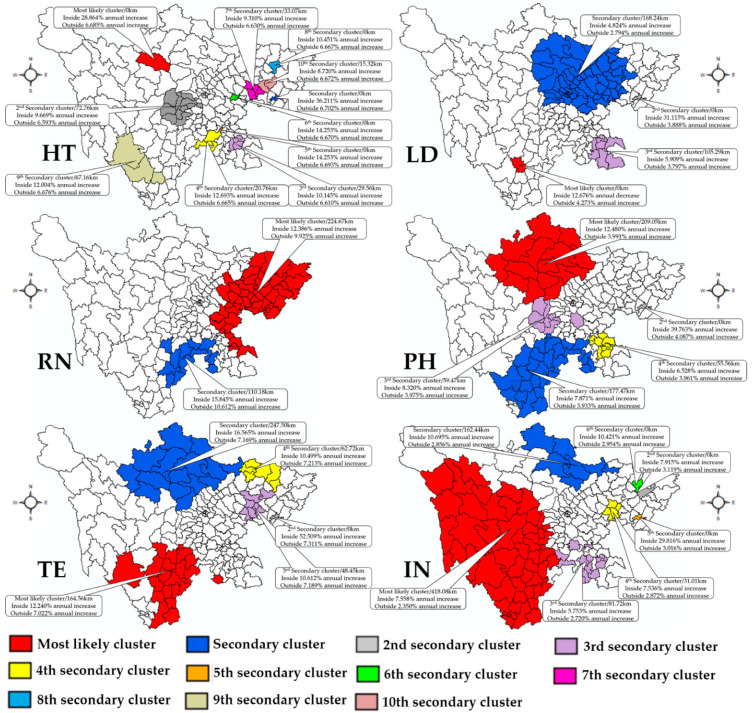
Higher spatial variation in temporal trends. HT, health technician; LD, licensed doctor; RN, registered nurse; PH, pharmacist; TE, technologist; IN, intern.

The results of the space–time scan conducted by absolute number are shown in Figure 6 and Figure 7. From 2009 to 2019, the statistic detected the most likely clusters and secondary clusters and other clusters of the different types of healthcare professionals, which demonstrated the different growth between the inside and the outside. Figure 6 displays the clusters which showed a lower growth rate comparing with surrounding regions, while Figure 7 demonstrates the regions with higher growth rate in contrast to surrounding regions. Taking HT as an example, compared with the surrounding area, the most likely clusters with lower growth were detected in the capital region, while the most likely clusters with higher growth were found in a specific county Maerkangshi. More information regarding the most likely clusters and other clusters is shown in Table 2.

## 4. Discussion

This research study performed a comparative spatio-temporal analysis of the healthcare professionals (HT, LD, RN, pharmacist, technologist, and interns) in Sichuan province of China, which produced much evidence for formulating policies and strategies of healthcare professional attraction and retention.

Firstly, as for the total quantity of health technicians in Sichuan, its absolute number increased from 303,051 to 603,073 in eleven years and the average annual growth rate was 7.12%. According to the results of our additional analysis on other provincial units, the average annual growth rate of China was 6.25% during the research period, while the number was 5.43%, 6.20% and 7.24%, respectively; in Beijing (Eastern China), Hubei (Middle China) and Ningxia (Western China), the temporal trends for the subtypes of HT were also similar. By the end of 2019, the number of HT in Sichuan province has accounted for 5.90% of the total number of HT in China, indicating that the scarcity of healthcare professionals in Sichuan has gradually eased. The Chinese government have issued a series of policies for the attraction and retention of healthcare professionals since the New Medical Reform such as *the long-term medical personnel development plan of China (2011–2020), 13th Five-Year Plan for medical professionals.* This could be regarded as the main reason for the improvement of quantity of healthcare professionals. The numbers of LD and RN per 1000 of the population were 2.34 and 2.77, respectively. According to data from the World Bank WDI Database in 2018, the number of physicians per 1000 people in high-income countries is 3.0 [35,36]. Sichuan still needs to take various measures in educating and attracting doctors. The overall team-building of nursing human resources in Sichuan Province was better, but the ratio of doctors and nurses was far from what is required (1:2 in the trial draft of organization budgeting principles on Comprehensive Hospital personnel issued by the National Health Commission of the PRC [37]).

Secondly, Sichuan is facing severe problems in the distribution of qualified healthcare professionals, which echoes the unbalanced distribution of healthcare professionals across the provincial units in China [38,39,40]. There are several reasons for this situation, including the unbalanced development among regions, such as the tension between doctors and patients [41,42], and the huge economic and social differences between regions.

The global Moran’s I of the number of all types of healthcare professionals per 1000 people in Sichuan reached the 0.001 significance level throughout the research period, demonstrating the spatial disparities in the distribution of healthcare professionals. While it is important to note that different types of healthcare professionals displayed relatively different spatio-temporal characteristics, while a similar distribution pattern was observed in the distribution of LD, RN, pharmacists and technologists in 2019. That is, the situation of uneven distribution of these types of healthcare professionals was narrowed down, but a shortage of these types of healthcare professionals was still seen in the western and eastern Sichuan. Compared with the plain area, the plateau and mountain areas were in relative lack of licensed doctors and registered nurses. In other words, even though a lot of policy preferences have been given to some western units, while these policies owned limited effects in allocating health workforce to the plateau and mountain areas. At the same time, it is hard for citizens in these areas to access sufficient medical and health services [43,44]. Understandably, there are large disparities between the western mountainous region (minority-group regions) and capital areas due to the instinct difference in terms of development, but the most critical situation was happening in the northeastern region and southeastern region such as Bazhong, Dazhou, Guangan, Nanchong and Leshan, which owned a lower number of healthcare professionals per 1000 people compared to the most underprivileged region. This could be explained by the transportation access of these cities with rich regions such as Chengdu and Mianyang. The convenient transportation give healthcare professionals in these regions enough time to travel from work to home. They are more willing to be a staff member in rich regions for the salary and benefits comparing with the healthcare professionals in distant western regions.

Interestingly, interns displayed unique characteristics compared with other types of healthcare professionals. The units located in Qinghai Tibet Plateau and Chengdu (capital of Sichuan) had the highest number per 1000 of the population, as shown in the hierarchical map and cluster map. This could be attributed to the lack of qualified healthcare professionals in these regions that the health institutions could only turn to recruit more interns. Apart from that, the government is trying to ease the situation of the shortage of healthcare professionals by requesting the interns to serve the western region first.

In addition to that, there are huge differences between the subtypes of healthcare professionals, which may be attributable to the different supply channel and training requirements of different subtypes of healthcare professionals. This appeals to subtype-specific health workforce planning and allocation policies for the balance of health workforce distribution.

Thirdly, in terms of the growth rate of all healthcare professionals, there are two main findings: firstly, the poorest region of healthcare professionals per 1000 people showed the higher growth rate, while the highest region of that showed the lower growth rate. Chengdu had a most likely cluster of lower growth rates compared to the surrounding areas in all healthcare professionals. Some regions in the northeastern region and southeastern region showed increased rates such as the HT, RN and interns. This could be contributing to the number base of all different healthcare professionals. Secondly, there are differences in growth rate for different healthcare professionals in the western region (minority-group regions). Specifically, the growth rate of pharmacists, technologists and interns was much higher than in the surrounding region, while the growth rate of HT, LD, and RN is not as fast as others.

To conclude, the spatial disparities in access to healthcare professionals remain huge in Sichuan. Based on the results of these studies, we believe that the county-level disparities may deserve consideration by policy makers and health leaders when making related policies. Our study highlights the need for and importance of improving access to healthcare professionals in rural and remote areas, especially those units located in Tsinling Mountains, Yunnan-Guizhou Plateau, Hengduan Mountains, and the transition zone of mountains and basins. Educating more medical students in these regions can be one of the first things, while a balanced skill mix of different types of healthcare professionals is also essential for delivering high-quality and efficient health services. The findings of this study also have the merit of demonstrating disparities in healthcare professional distribution in other western provincial units, such as Gansu and Yunnan.

Concerning the limitations of the research, this study only used the number of healthcare professionals per 1000 of the population to represent the accessibility to healthcare professionals in one unit, which limits the patients’ access within a municipality. Additionally, only the case of Sichuan is not enough to extend the research findings to the whole country. Moreover, the data were not precise enough to explore the spatial accessibility of healthcare-professional resources, few studies are suggested to include the distance data of people and healthcare facilities. More research using other indicators and targeting other units are encouraged to delve into this important area.

## 5. Conclusions

Most previous studies paid attention to the province-level distribution of healthcare professionals, while this study zooms into the county-level; thus, a new and nuanced picture of the spatial disparities in access to healthcare professionals was drawn. According to the temporal and spatial analysis conducted in our study, there are still many tremendous obstacles in dealing with the spatial disparities in access to healthcare professionals in order to achieve health equity in Sichuan, policy makers should pay more attention to rural and remote areas, especially those units located in Tsinling Mountains, Yunnan-Guizhou Plateau, Hengduan Mountains and the transition zone of mountains and basins. The findings may deserve considerations in making region-oriented policies in educating and attracting more healthcare professionals to the disadvantaged areas.

## Figures and Tables

**Table 1:** Global spatial autocorrelation analysis and significance test results of the number of healthcare professionals per 1000 people.

**Year**	**HT**		**LD**		**RN**	
	**Moran’s I**	***p* Value**	**Moran’s I**	***p* Value**	**Moran’s I**	***p* Value**
2009	0.423706 ***	0.000010	0.405813 ***	0.000010	0.410237 ***	0.000010
2010	0.416124 ***	0.000010	0.406026 ***	0.000010	0.407913 ***	0.000010
2011	0.391384 ***	0.000010	0.405055 ***	0.000010	0.405743 ***	0.000010
2012	0.391384 ***	0.000010	0.397521 ***	0.000010	0.379301 ***	0.000010
2013	0.372937 ***	0.000010	0.411781 ***	0.000010	0.385633 ***	0.000010
2014	0.358038 ***	0.000010	0.398195 ***	0.000010	0.360035 ***	0.000010
2015	0.344185 ***	0.000010	0.381742 ***	0.000010	0.351074 ***	0.000010
2016	0.345551 ***	0.000010	0.402015 ***	0.000010	0.346748 ***	0.000010
2017	0.347294 ***	0.000010	0.406406 ***	0.000010	0.352470 ***	0.000010
2018	0.294261 ***	0.000010	0.352636 ***	0.000010	0.293614 ***	0.000010
2019	0.313754 ***	0.000010	0.367173 ***	0.000010	0.316865 ***	0.000010
**Year**	**PH**		**TE**		**IN**	
	**Moran** **’** **s I**	***p*** **Value**	**Moran** **’** **s I**	***p*** **Value**	**Moran** **’** **s I**	***p*** **Value**
2009	0.522996 ***	0.000010	0.344224 ***	0.000010	0.647758 ***	0.000010
2010	0.520049 ***	0.000010	0.368554 ***	0.000010	0.617869 ***	0.000010
2011	0.532507 ***	0.000010	0.362150 ***	0.000010	0.612072 ***	0.000010
2012	0.469997 ***	0.000010	0.289329 ***	0.000010	0.620311 ***	0.000010
2013	0.517939 ***	0.000010	0.282955 ***	0.000010	0.544234 ***	0.000010
2014	0.525362 ***	0.000010	0.306172 ***	0.000010	0.549515 ***	0.000010
2015	0.473459 ***	0.000010	0.311680 ***	0.000010	0.587813 ***	0.000010
2016	0.483436 ***	0.000010	0.295715 ***	0.000010	0.637788 ***	0.000010
2017	0.487016 ***	0.000010	0.319444 ***	0.000010	0.675645 ***	0.000010
2018	0.423995 ***	0.000010	0.271670 ***	0.000010	0.710535 ***	0.000010
2019	0.423461 ***	0.000010	0.277780 ***	0.000010	0.763703 ***	0.000010

* means 10% level of significance; ** means 5% level of significance; *** means 1% level of significance. HT, health technician; LD, licensed doctor; RN, registered nurse; PH, pharmacist; TE, technologist; IN, intern.

**Table 2:** The space–time results of all types of healthcare professionals.

Types	Cluster Type	Center	Areas	Coordinates	Radius (Km)	RR	Inside Time Trend	Outside Time Trend	*p*-Value
HT	Most likely cluster	Jinjiangqu (Chengdu)	6	30.67 N, 104.08 E	18.28	3.93	3.761% annual increase	6.765% annual increase	0.001
HT	Secondary cluster	Maerkangshi (Aba)	1	31.90 N, 102.22 E	0	1.33	28.864% annual increase	6.685% annual increase	0.001
HT	2nd secondary cluster	Qianfengqu (Guangan)	1	30.33 N, 106.49 E	0	0.28	36.211% annual increase	6.702% annual increase	0.001
HT	3rd secondary cluster	Baoxingxian (Yaan)	11	30.37 N, 102.82 E	72.76	1.01	9.669% annual increase	6.593% annual increase	0.001
HT	4th secondary cluster	Naxiqu (Luzhou)	4	28.77 N, 105.37 E	29.56	1.22	10.145% annual increase	6.610% annual increase	0.001
HT	Please see other clusters in Figure 6 and Figure 7								
LD	Most likely cluster	Xichangshi (Liangshan)	1	27.90 N, 102.27 E	0	3.49	12.676% annual decrease	4.273% annual increase	0.001
LD	Secondary cluster	Anzhouqu (Mianyang)	58	31.53 N, 104.57 E	168.24	1.58	4.824% annual increase	2.794% annual increase	0.001
LD	2nd secondary cluster	Qianfengqu (Guangan)	1	30.33 N, 106.49 E	0	0.27	31.115% annual increase	3.888% annual increase	0.001
LD	3rd secondary cluster	Bazhouqu (Bazhong)	6	31.85 N, 106.77 E	62.56	0.80	1.408% annual increase	4.011% annual increase	0.001
LD	4th secondary cluster	Gulinxian (Luzhou)	8	28.05 N, 105.82 E	105.29	0.79	5.909% annual increase	3.797% annual increase	0.001
RN	Most likely cluster	Jinjiangqu (Chengdu)	4	30.67 N, 104.08 E	18.28	4.70	5.672% annual increase	11.244% annual increase	0.001
RN	Secondary cluster	Linshuixian (Guangan)	49	30.33 N, 106.93 E	224.67	0.51	12.386% annual increase	9.925% annual increase	0.001
RN	2nd secondary cluster	Xichangshi (Liangshan)	1	27.90 N, 102.27 E	0	2.51	4.936% annual increase	10.759% annual increase	0.001
RN	3rd secondary cluster	Leiboxian (Liangshan)	12	28.27 N, 103.57 E	110.18	0.46	15.845% annual increase	10.612% annual increase	0.001
PH	Most likely cluster	Jinjiangqu (Chengdu)	6	30.67 N, 104.08 E	18.28	3.95	1.043% annual increase	4.193% annual increase	0.001
PH	Secondary cluster	Jiangyoushi (Mianyang)	1	31.78 N, 104.75 E	0	1.62	3.027% annual decrease	4.194% annual increase	0.001
PH	2nd secondary cluster	Hongyuanxian (Aba)	15	32.80 N, 102.55 E	209.05	0.77	12.480% annual increase	3.991% annual increase	0.001
PH	3rd secondary cluster	Jinyangxian (Liangshan)	27	27.70 N, 103.25 E	177.47	0.51	7.871% annual increase	3.933% annual increase	0.001
PH	4th secondary cluster	Qianfengqu (Guangan)	1	30.33 N, 106.49 E	0	0.25	39.763% annual increase	4.087% annual increase	0.001
PH	Please see other clusters in Figure 6 and Figure 7								
TE	Most likely cluster	Wuhouqu (Chengdu)	3	30.65 N, 104.05 E	3.63	5.74	2.312% annual increase	7.547% annual increase	0.001
TE	Secondary cluster	Zhaojuexian (Liangshan)	26	28.02 N, 102.85 E	164.56	0.75	12.240% annual increase	7.022% annual increase	0.001
TE	2nd secondary cluster	Ruoergaixian (Aba)	15	33.58 N, 102.95 E	247.50	1.08	16.565% annual increase	7.169% annual increase	0.001
TE	3rd secondary cluster	Qianfengqu (Guangan)	1	30.33 N, 106.49 E	0	0.33	52.509% annual increase	7.311% annual increase	0.001
TE	4th secondary cluster	Longchangshi (Neijiang)	3	29.35 N, 105.28 E	33.89	0.94	3.207% annual increase	7.434% annual increase	0.001
TE	Please see other clusters in Figure 6 and Figure 7								
IN	Most likely cluster	Wenjiangqu (Chengdu)	16	30.70 N, 103.83 E	44.48	1.86	1.481% annual decrease	3.938% annual increase	0.001
IN	Secondary cluster	Derongxian (Ganzi)	52	28.72 N, 99.28 E	418.08	1.36	7.558% annual increase	2.350% annual increase	0.001
IN	2nd secondary cluster	Jiuzhaigouxian (Aba)	7	33.27 N, 104.23 E	162.44	1.66	10.695% annual increase	2.856% annual increase	0.001
IN	3rd secondary cluster	Pingchangxian (Bazhong)	1	31.57 N, 107.10 E	0	0.72	7.915% annual decrease	3.119% annual increase	0.001
IN	4th secondary cluster	Cuipingqu (Yibin)	20	28.77 N, 104.62 E	81.72	0.89	5.753% annual increase	2.720% annual increase	0.001
IN	Please see other clusters in Figure 6 and Figure 7								

## Data Availability

Data is contained within the article or Supplementary Material. The data presented in this study are available in [Tables S2–S4].

## Supplementary Materials

The following are available online at https://www.mdpi.com/article/10.3390/healthcare9081053/s1, Table S1: The number of healthcare professionals in county-level units in Sichuan; Table S2: The population of county-level units in Sichuan province; Table S3: The number of healthcare professionals at the municipal level in Sichuan; Table S4: Growth in the number of healthcare professionals per 1000 people in Sichuan; Table S5: The p-value results for chi-square linear by linear association test.
