# Supplementary material for: Spatial Disparities in Access to Healthcare Professionals in Sichuan: Evidence from County-Level Data
Source: Healthcare (Basel). 2021 Aug 16;9(8):1053. doi: 10.3390/healthcare9081053 (PMC8394835; doi:10.3390/healthcare9081053)
Supplement: Supplementary file 1 [file healthcare-09-01053-s001.zip › healthcare-1239674 Supplmental materials/Table S1.pdf]

Table S1 Growth of the number of health workforce per 1000 population in China

| region | HT<br>09 $\underline{\text{aunal average growth rate}}$ 17 | Growth rate rank | LD<br>09 $\underline{\text{annual average growth rate}}$ 17 | Growth rate rank | RN<br>09 $\underline{\text{annual average growth rate}}$ 17 | Growth rate rank |
|---|---|---|---|---|---|---|
| Beijing | 12.92 $\underline{-1.66\%}$ 11.30 | 30 | 5.04 $\underline{-1.97\%}$ 4.30 | 30 | 4.95 $\underline{-0.38\%}$ 4.80 | 30 |
| Tianjin | 6.90 $\underline{-0.74\%}$ 6.50 | 29 | 2.80 $\underline{0.92\%}$ 2.60 | 29 | 2.34 $\underline{0.83\%}$ 2.50 | 29 |
| Hebei | 3.71 $\underline{5.51\%}$ 5.70 | 18 | 1.72 $\underline{5.30\%}$ 2.60 | 9 | 1.04 $\underline{9.18\%}$ 2.10 | 13 |
| Shanxi | 5.38 $\underline{1.99\%}$ 6.30 | 28 | 2.45 $\underline{0.25\%}$ 2.50 | 27 | 1.65 $\underline{5.85\%}$ 2.60 | 25 |
| Neimenggu | 5.50 $\underline{3.24\%}$ 7.10 | 24 | 2.82 $\underline{-0.09\%}$ 2.80 | 28 | 1.44 $\underline{8.67\%}$ 2.80 | 17 |
| Liaoning | 5.32 $\underline{2.92\%}$ 6.70 | 26 | 2.25 $\underline{1.82\%}$ 2.60 | 25 | 1.99 $\underline{4.82\%}$ 2.90 | 28 |
| Jilin | 4.87 $\underline{3.06\%}$ 6.20 | 25 | 2.21 $\underline{2.05\%}$ 2.60 | 24 | 1.56 $\underline{6.07\%}$ 2.50 | 24 |
| Heilongjiang | 4.56 $\underline{3.70\%}$ 6.10 | 22 | 1.93 $\underline{2.22\%}$ 2.30 | 22 | 1.45 $\underline{6.50\%}$ 2.40 | 23 |
| Shanghai | 9.48 $\underline{-2.57\%}$ 7.70 | 31 | 3.79 $\underline{-3.71\%}$ 2.80 | 31 | 3.73 $\underline{-0.79\%}$ 3.50 | 31 |
| Jiangsu | 4.16 $\underline{6.34\%}$ 6.80 | 11 | 1.69 $\underline{6.03\%}$ 2.70 | 6 | 1.50 $\underline{9.05\%}$ 3.00 | 14 |
| Zhejiang | 5.65 $\underline{4.61\%}$ 8.10 | 20 | 2.41 $\underline{3.61\%}$ 3.20 | 20 | 1.87 $\underline{7.36\%}$ 3.30 | 22 |
| Anhui | 3.07 $\underline{6.29\%}$ 5.00 | 13 | 1.25 $\underline{5.37\%}$ 1.90 | 8 | 1.03 $\underline{9.95\%}$ 2.20 | 10 |
| Fujian | 3.74 $\underline{5.86\%}$ 5.90 | 15 | 1.57 $\underline{3.70\%}$ 2.10 | 19 | 1.37 $\underline{8.34\%}$ 2.60 | 19 |
| Jiangxi | 3.25 $\underline{5.79\%}$ 5.10 | 17 | 1.28 $\underline{4.35\%}$ 1.80 | 15 | 1.16 $\underline{8.93\%}$ 2.30 | 15 |
| Shandong | 4.39 $\underline{5.82\%}$ 6.90 | 16 | 1.86 $\underline{4.28\%}$ 2.60 | 16 | 1.48 $\underline{8.77\%}$ 2.90 | 16 |
| Henan | 3.38 $\underline{7.66\%}$ 6.10 | 7 | 1.42 $\underline{0.21\%}$ 2.30 | 4 | 1.05 $\underline{11.45\%}$ 2.50 | 5 |
| Hubei | 4.02 $\underline{6.79\%}$ 6.80 | 8 | 1.59 $\underline{5.82\%}$ 2.50 | 7 | 1.42 $\underline{10.25\%}$ 3.10 | 9 |
| Hunan | 3.62 $\underline{6.74\%}$ 6.10 | 9 | 1.51 $\underline{6.51\%}$ 2.50 | 2 | 1.19 $\underline{9.72\%}$ 2.50 | 11 |
| Guangdong | 5.04 $\underline{2.83\%}$ 6.30 | 27 | 1.93 $\underline{2.22\%}$ 2.30 | 22 | 1.83 $\underline{5.46\%}$ 2.80 | 26 |
| Guangxi | 3.32 $\underline{8.12\%}$ 6.20 | 5 | 1.28 $\underline{6.28\%}$ 2.10 | 3 | 1.21 $\underline{10.55\%}$ 2.70 | 8 |
| Hainan | 4.30 $\underline{5.30\%}$ 6.50 | 19 | 1.60 $\underline{4.06\%}$ 2.20 | 18 | 1.73 $\underline{7.56\%}$ 3.10 | 21 |
| Chongqing | 3.05 $\underline{9.27\%}$ 6.20 | 2 | 1.36 $\underline{6.20\%}$ 2.20 | 5 | 0.97 $\underline{14.17\%}$ 2.80 | 2 |
| Sichuan | 3.37 $\underline{8.35\%}$ 6.40 | 4 | 1.54 $\underline{5.14\%}$ 2.30 | 11 | 1.01 $\underline{13.59\%}$ 2.80 | 3 |
| Guizhou | 2.37 $\underline{13.00\%}$ 6.30 | 1 | 1.01 $\underline{9.58\%}$ 2.10 | 1 | 0.78 $\underline{16.79\%}$ 2.70 | 1 |
| Yunnan | 3.02 $\underline{8.73\%}$ 5.90 | 3 | 1.35 $\underline{5.04\%}$ 2.00 | 12 | 1.04 $\underline{12.67\%}$ 2.70 | 4 |
| Xizang | 3.49 $\underline{4.33\%}$ 4.90 | 21 | 1.57 $\underline{4.89\%}$ 2.30 | 13 | 0.69 $\underline{8.24\%}$ 1.30 | 20 |
| Shaanxi | 4.46 $\underline{7.74\%}$ 8.10 | 6 | 1.81 $\underline{3.59\%}$ 2.40 | 21 | 1.42 $\underline{11.12\%}$ 3.30 | 6 |
| Gansu | 3.38 $\underline{6.51\%}$ 5.60 | 10 | 1.39 $\underline{5.29\%}$ 2.10 | 10 | 0.98 $\underline{10.64\%}$ 2.20 | 7 |
| Qinghai | 4.43 $\underline{5.89\%}$ 7.00 | 14 | 1.88 $\underline{4.14\%}$ 2.60 | 17 | 1.44 $\underline{8.67\%}$ 2.80 | 17 |
| Ningxia | 4.48 $\underline{6.29\%}$ 7.30 | 12 | 1.91 $\underline{4.42\%}$ 2.70 | 14 | 1.55 $\underline{9.48\%}$ 3.20 | 12 |
| Xinjiang | 5.47 $\underline{3.31\%}$ 7.10 | 23 | 2.20 $\underline{1.61\%}$ 2.50 | 26 | 1.92 $\underline{5.29\%}$ 2.90 | 27 |
| SUM | 4.15 $\underline{5.77\%}$ 6.50 | - | 1.75 $\underline{4.03\%}$ 2.40 | - | 1.39 $\underline{8.65\%}$ 2.70 | - |

Unit: Persons
