# Supplementary material for: Spatial Disparities in Access to Healthcare Professionals in Sichuan: Evidence from County-Level Data
Source: Healthcare (Basel). 2021 Aug 16;9(8):1053. doi: 10.3390/healthcare9081053 (PMC8394835; doi:10.3390/healthcare9081053)
Supplement: Supplementary file 1 [file healthcare-09-01053-s001.zip › healthcare-1239674 Supplmental materials/Table S5.pdf]

Table S5 The p-value results for chi-square linear by linear association test

Region	Health technicians		Licensed doctor		Registered nurses		Pharmacist		Technologist		Interns	
	09-13	13-17	09-13	13-17	09-13	13-17	09-13	13-17	09-13	13-17	09-13	13-17
Chengdu	0.046	0.047	0.046	0.048	0.046	0.046	0.047	0.047	0.046	0.047	0.071	0.288
Zigong	0.050	0.046	0.050	0.112	0.050	0.046	0.056	0.050	0.051	0.047	0.064	0.074
Panzhihua	0.053	0.059	0.071	0.279	0.050	0.058	0.142	0.051	0.066	0.074	0.193	0.391
Luzhou	0.049	0.051	0.047	0.055	0.048	0.047	0.046	0.079	0.053	0.202	0.147	0.107
Deyang	0.047	0.046	0.048	0.060	0.046	0.046	0.052	0.060	0.046	0.047	0.061	0.221
Mianyang	0.048	0.048	0.052	0.077	0.046	0.046	0.097	0.462	0.056	0.071	0.076	0.060
Guangyuan	0.050	0.047	0.138	0.860	0.049	0.046	0.049	0.442	0.049	0.050	0.066	0.101
Suining	0.047	0.050	0.047	0.116	0.047	0.048	0.050	0.058	0.049	0.060	0.605	0.065
Neijiang	0.059	0.059	0.072	0.081	0.047	0.048	0.226	0.047	0.074	0.469	0.194	0.075
Leshan	0.047	0.049	0.056	0.166	0.046	0.046	0.077	0.223	0.050	0.050	0.934	0.776
Nanchong	0.046	0.046	0.053	0.046	0.052	0.059	0.053	0.049	0.063	0.046	0.050	0.111
Meishan	0.055	0.059	0.075	0.373	0.052	0.049	0.063	0.106	0.080	0.061	0.074	0.482
Yibin	0.052	0.051	0.072	0.106	0.048	0.047	0.080	0.048	0.061	0.047	0.054	0.115
Guangan	0.049	0.046	0.048	0.046	0.050	0.046	0.060	0.050	0.070	0.057	0.055	0.079
Dazhou	0.050	0.068	0.053	0.424	0.050	0.053	0.090	0.162	0.053	0.066	0.119	0.059
Yaan	0.062	0.047	0.058	0.066	0.052	0.047	0.122	0.111	0.068	0.050	0.708	0.071
Bazhong	0.050	0.053	0.046	0.731	0.052	0.046	0.056	0.050	0.123	0.058	0.790	0.107
Ziyang	0.046	0.149	0.046	0.091	0.047	0.875	0.058	0.232	0.047	0.334	0.054	0.070
Abazhou	0.071	0.047	0.418	0.054	0.053	0.046	0.068	0.065	0.075	0.049	0.173	0.055
Ganzizhou	0.078	0.046	0.828	0.336	0.054	0.046	0.067	0.343	0.064	0.059	0.242	0.056
Liangshanzhou	0.054	0.046	0.082	0.081	0.082	0.046	0.072	0.049	0.078	0.046	0.056	0.059
SUM	0.047	0.046	0.046	0.051	0.046	0.046	0.047	0.046	0.049	0.048	0.069	0.080
